# Estimating the basic reproductive ratio for the Ebola outbreak in Liberia and Sierra Leone

**DOI:** 10.1186/s40249-015-0043-3

**Published:** 2015-02-24

**Authors:** Adnan Khan, Mahim Naveed, Muhammad Dur-e-Ahmad, Mudassar Imran

**Affiliations:** Department of Mathematics, Lahore University of Management Sciences, Lahore, Pakistan; Department of Mathematics, State University of New York at Plattsburgh, Plattsburgh, NY USA; Department of Mathematics, Waterloo University, Waterloo, ON Canada; Department of Mathematics and Natural Sciences, GULF University for Science and Technology, Mubarak Al-Abdullah, West Mishref Kuwait

**Keywords:** Epidemiology, Ebola virus disease, Transmission model, Basic reproductive ratio

## Abstract

**Background:**

Ebola virus disease has reemerged as a major public health crisis in Africa, with isolated cases also observed globally, during the current outbreak.

**Methods:**

To estimate the basic reproductive ratio R_0_, which is a measure of the severity of the outbreak, we developed a SEIR (susceptible-exposed-infected-recovered) type deterministic model, and used data from the Centers for Disease Control and Prevention (CDC), for the Ebola outbreak in Liberia and Sierra Leone. Two different data sets are available: one with raw reported data and one with corrected data (as the CDC suspects under-reporting).

**Results:**

Using a deterministic ordinary differential equation transmission model for Ebola epidemic, the basic reproductive ratio R_0_ for Liberia resulted to be 1.757 and 1.9 for corrected and uncorrected case data, respectively. For Sierra Leone, R_0_ resulted to be 1.492 and 1.362 for corrected and uncorrected case data, respectively. In each of the two cases we considered, the estimate for the basic reproductive ratio was initially greater than unity leading to an epidemic outbreak.

**Conclusion:**

We obtained robust estimates for the value of R_0_ associated with the 2014 Ebola outbreak, and showed that there is close agreement between our estimates of R_0_. Analysis of our model also showed that effective isolation is required, with the contact rate in isolation less than one quarter of that for the infected non-isolated population, and that the fraction of high-risk individuals must be brought to less than 10% of the overall susceptible population, in order to bring the value of R_0_ to less than 1, and hence control the outbreak.

**Electronic supplementary material:**

The online version of this article (doi:10.1186/s40249-015-0043-3) contains supplementary material, which is available to authorized users.

## Multilingual abstracts 

Please see Additional file 1 for translations of the abstract into the six official working languages of the United Nations.

## Background

Ebola virus disease (EVD), named after the Ebola River in Zaire, is known to be a highly contagious disease with a high mortality rate [1,2]. Previously known as Ebola hemorrhagic Fever, EVD has a number of different strains. Originating in Sudan and Zaire in 1976, there has been a number of over the years [1,2]. From 1976 to 2008, the total case fatality rate for EVD victims was 79% [3]. The ongoing outbreak of EVD is affecting multiple countries in Central and Western Africa [2]. Beginning in December 2013 in West Africa, precisely Guinea, the EVD outbreak spread to Sierra Leone, Liberia and Nigeria with fatality rates of 73.2%, 43.0%, 52.5% and 33.3% respectively [4]. In May 2014, the second outbreak was confirmed in nearby regions, including Sierra Leone and Liberia [3]. As of October 14th 2014, 4,555 Ebola deaths have been reported in these countries, including one death in the United States [5].

EVD is a viral infection caused by a virus of the family *Filoviridae*, genus *Ebolavirus*. There are five identified subspecies of the Ebolavirus. Four of the five subspecies: i) Ebolavirus (*Zaire ebolavirus*), EBOV ii) Sudan virus (*Sudan ebolavirus*), SUDV, iii) Bundibugyo virus (*Bundibugyo ebolavirus*), BDBV and iv) Ta Forest virus (*Ta Forest ebolavirus*), TAFV, have caused disease in humans. The fifth, called the Reston virus (*Reston ebolavirus*), has caused disease in nonhuman primates [2]. The primary source of Ebola virus is considered to be fruit bats of the Pteropodidae family, with monkeys, gorillas, and chimpanzees believed to further transmit the disease [4].

Ebola is transmitted through direct contact with the skin, blood or bodily fluids of an infected individual or animal and with contaminated objects [2,6]. Individuals who take care of an infected person or bury someone who has died from the disease can also acquire the virus [2]. There is evidence that health care workers and relatives may become infected after contact with a patient or patient’s bodily fluids. It must be noted that a recovered individual cannot spread the virus but the Ebola virus has been found to remain in semen for up to three months. Therefore, abstinence from sex is recommended for at least this period of time [6]. From the outbreaks since 1976, it has been observed that the Ebola virus cannot naturally transmit through air, water, or food like influenza or diarrheal diseases [2,4]. Furthermore, individuals suffering from EVD do not infect other individuals during the incubation period, which can last between two and twenty-one days [4]. Common symptoms of EVD include fever, myalgia, malaise, sore throat, chest pain, red eyes, hiccups, rash, weakness, severe headaches, joint and muscle pain, diarrhea, vomiting, stomach pain, dehydration, dry and hacking cough, and loss of appetite. These symptoms typically start two days to three weeks after acquiring EVD. As the infection spreads, the body undergoes severe blood loss and coagulation abnormalities. Ultimately, the liver, kidney, and micro vascular endothelial cells (capillary walls) become infected, leading to compromise of vascular integrity. If not diagnosed and treated, death usually occurs in the second week of symptoms, and is usually caused by massive blood loss [4].

Recovery from Ebola is dependent on good supportive clinical care and the infected individual's immune response. Fortunate individuals who recover from EVD develop antibodies that last for at least 10 years [6]. These individuals may still experience weakness, fatigue, headaches, hair loss, hepatitis, sensory changes, and inflammation of organs [4].

Over the years, a few models for EVD have been studied and analyzed. Thomas E. Sutto has used an exponential fitting of data provided by the Centers of Disease Control and Prevention (CDC) and the World Health Organization (WHO) to develop formulae that best fit infection rate totals [7]. Similarly, Camacho et al. depicted the potential for large EVD outbreaks by fitting a mathematical model to time series, estimating epidemiological factors responsible for disease transmission [8]. Another significant contribution estimated parameters from daily incidence and mortality time series for the 1995 Congo Ebola outbreak [9]. Similarly, Chowell et al. used epidemic modeling to estimate the number of secondary cases generated by an index case in the absence of control interventions [10]. In another study, Chowell et al. carried out a comparative review of mathematical models of the spread and control of Ebola [11]. Legrand et al. have previously studied transmission dynamics in order to derive a relationship between the hospitalization rate and epidemic size [12].

Apart from mathematical modeling of Ebola, noteworthy non-mathematical contributions have also been made. Recently, Tambo and Xiao-Nong examined research, prevention, detection, and management related issues of the Ebola outbreak and reflected upon the major gaps in frontline and airport Ebola control and containment, providing possible structured opportunities to the public [13]. Lai et al. provided insight into non-mutable host cell therapeutic agents targeting different steps of the life cycle of the Ebola virus [14]. Tambo et al. proposed surveillance response systems for controlling the Ebola outbreak shedding light on the use of early warnings, critical human resources development, and methods to enhance tracking and managing challenges and urging further development in new drug discovery and vaccines [15]. Tambo also shed light on non-conventional humanitarian interventions on Ebola in another recent study [16].

None of the above mentioned models have used the aspect of two susceptible populations: high-risk and low-risk. The model we present in this paper explains different aspects of the disease dynamics. In the following section we explore the formulation of our model. Then we explain the data fitting technique and present our results.

The purpose of our study is to estimate the basic reproductive ratio (R_0_) for the EVD outbreak in Liberia and Sierra Leone. Using data obtained from the CDC for the period of May 1st, 2014 up until October 1st, 2014, we present a deterministic SEIR type model for the transmission dynamics of the Ebola virus to estimate R_0_. We also present an illustration of the required disease control scenario to achieve R_0_ < 1.

## Methods

### Mathematical model formulation

We base our study on a deterministic ordinary differential equations (ODE) epidemic model in which the population size is divided into six mutually exclusive compartments. The total population at any time instant t, denoted by *N(t)*, is the sum of individual populations in each compartment that includes low-risk susceptible individuals *S*_*L*_*(t)*, high-risk susceptible individuals *S*_*H*_*(t)*, exposed individuals *E(t)*, infected individuals *I(t)*, hospitalized individuals *H(t)*, and recovered individuals *R(t)* such that,

$$ N(t) = {S}_L(t) + {S}_H(t) + E(t) + I(t) + H(t) + R(t) $$

The high-risk susceptible population includes health-care workers and providers (including all front-line workers), relatives of infected individuals, and the people involved in burial processes. The rest of the susceptible population is considered to be at a low risk of acquiring EVD.

**Figure 1:**
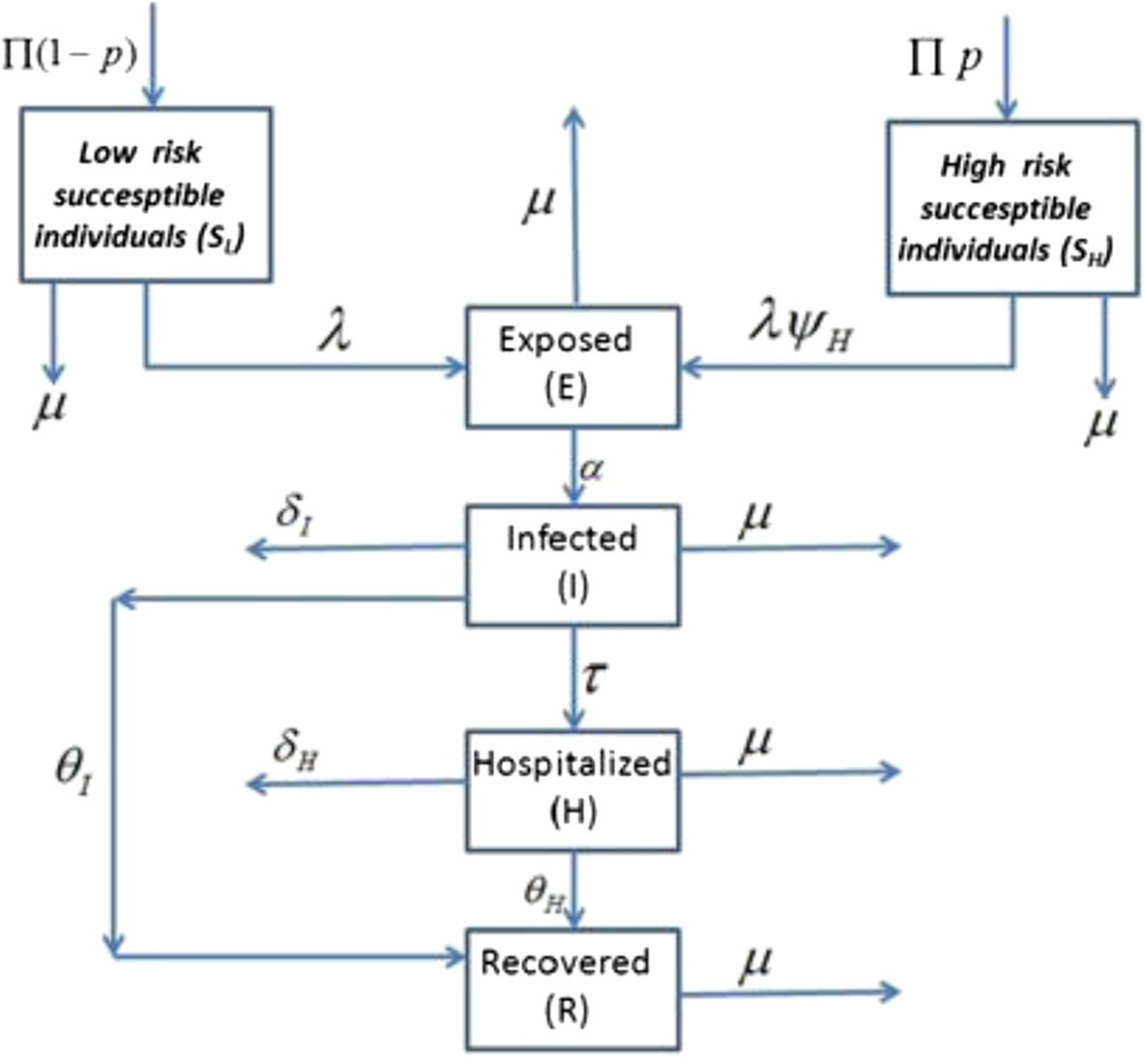
**Shows a flow diagram depicting the dynamics of the Ebola virus across all compartments.**

Since there is no vertical transmission of the infection, we assume that all newborns are susceptible. The susceptible population increases at a constant recruitment rate Π (all recruited individuals are assumed to be susceptible), and *p* is the fraction of recruited individuals who are at a high risk of acquiring the infection. Low-risk susceptible individuals acquire the infection at a rate *λ*. Furthermore, the susceptible population also decreases at the natural death rate *μ*. An increase in the high-risk population *S*_*H*_ means that there are more health-care workers and more people involved in the burial process. The rate at which infection is acquired by susceptible classes, also recognized as the force of infection, depends on the proportion of the infected and hospitalized individuals, taking into account the infectiousness of the hospitalized individuals (*η*). In our model, the force of infection is given by *λ*. The Exposed population increases after susceptible individuals acquire the infection at a rate *λ* from low-risk individuals or at a rate *ψ*_*H*_*λ* from high-risk individuals respectively. The population of infected individuals generated at a rate α decreases when these individuals go to a hospital at rate *τ*. It also decreases because of the natural death rate *μ*, and the disease-induced death rate *δ*_*I*_. Infected individuals recover from disease at rate of *θ*_*I*_. The number of hospitalized individuals is generated when infected individuals are hospitalized at a rate *τ*. It diminishes when individuals recover at a rate *θ*_*H*_, and die naturally or due to infection at rates *μ* and *δ*_*H*_, respectively. The flow diagram of Ebola model is shown in Figure 1.

The model in this study is given by the following system of non-linear differential equations:

1 $$ \begin{array}{l}\frac{d{S}_L}{dt}=\Pi \left(1-p\right)-\lambda {S}_L-\mu {S}_L\hfill \\ {}\begin{array}{cc}\hfill \frac{d{S}_H}{dt}=\Pi p-{\psi}_H\lambda {S}_H-\mu {S}_H\hfill & \hfill {\psi}_H>1\hfill \end{array}\hfill \\ {}\frac{dE}{dt}=\lambda \left({S}_L+{\psi}_H{S}_H\right)-\left(\alpha +\mu \right)E\hfill \\ {}\frac{dI}{dt}=\alpha E-\left(\tau +{\theta}_I+{\delta}_I+\mu \right)I\hfill \\ {}\frac{dH}{dt} = \tau I-\left({\theta}_H+{\delta}_H+\mu \right)H\hfill \\ {}\frac{dR}{dt}={\theta}_II+{\theta}_HH-\mu R\hfill \end{array} $$

where, $\lambda =\beta \frac{\left(I+\eta H\right)}{N}$ is called the force of infection.

All model parameters are summarized in Table 1 in Section 2.4.

**Table 1:** **Values of the parameters used in Model 1**

**Parameter**	**Description**	**Value**
***ψ***_***H***_	Modification parameter for infection rate of high-risk susceptible individuals	1.2-2
***δ***_***I***_	Disease-induced death rate of infected individuals	0.10
***δ***_***H***_	Disease-induced death rate of hospitalized individuals	0.5
***θ***_***I***_	Recovery rate of infected individuals	0.1
***θ***_***H***_	Recovery rate of hospitalized individuals	0.2
***α***	Rate at which latent individuals become infectious	0.1
***τ***	Hospitalization rate for infected individuals	0.16
**Π**	Recruitment rate	1.7
p	Fraction of the individuals at high-risk	0.2
β	Transmission rate of disease	Estimated
1/μ	Average life of human	63 years

### The basic reproductive ratio

The basic reproductive ratio (R_0_) is the number of individuals infected by a single infected individual during the infectious period in an entirely susceptible population [17]. As our model consists of multiple classes, the next generation operator is used to calculate R_0_ [17]. Because our population consists of a hospitalized class in addition to exposed and infected classes, our final *R*_*0*_ is the sum of *R*_*1*_ and *R*_*2*_ (resulting from the infectiousness linked to community and hospitals respectively).

We use the next generation matrix approach, as introduced by Diekmann et al., to calculate R_0_ [18]. Using differential equations associated with the exposed (*E*), infected (*I*), and hospitalized (*H*) compartments as stated below, we compute a function (*F*) for the rate of new infection terms entering, and another function (*V*) for the rate of transfer into and out of the exposed, infected, and hospitalized compartments by all possible means depicted in Model 1.

$$ E\hbox{'}=\lambda \left({S}_L+{\psi}_H{S}_H\right)-\left(\alpha +\mu \right)E,\kern2.75em {I}^{\hbox{'}}=\alpha E-\left(\tau +{\theta}_I+{\delta}_I+\mu \right)I\kern1.75em \mathrm{and}\kern1em H\hbox{'} = \tau I-\left({\theta}_H+{\delta}_H+\mu \right)H. $$

The matrices *F* (for the new infection terms) and *V* (of the transition terms) are given by,

$$ F=\left[\begin{array}{ccc}\hfill 0\hfill & \hfill \beta \Omega \hfill & \hfill \beta \upeta \Omega \hfill \\ {}\hfill 0\hfill & \hfill 0\hfill & \hfill 0\hfill \\ {}\hfill 0\hfill & \hfill 0\hfill & \hfill 0\hfill \end{array}\right]\kern2em \mathrm{and}\kern1.75em V=\left[\begin{array}{ccc}\hfill \alpha +\mu \hfill & \hfill 0\hfill & \hfill \beta \upeta \Omega \hfill \\ {}\hfill -\alpha \hfill & \hfill \uptau +{\uptheta}_{\mathrm{I}}+{\updelta}_{\mathrm{I}}+\upmu \hfill & \hfill 0\hfill \\ {}\hfill 0\hfill & \hfill -\uptau \hfill & \hfill {\mathrm{K}}_3\hfill \end{array}\right]. $$

where, $$ {K}_3={\theta}_H+{\delta}_H+\mu, \kern1em \mathrm{and}\kern1em \varOmega \kern0.5em =\kern0.75em \frac{\Pi \left(1-p\right)+{\psi}_Hp}{\mu }. $$

Reproductive ratio R_0_ is then given as

$$ {\mathrm{R}}_0=\rho \left(F{V}^{-1}\right) $$

where *ρ* is the spectral radius (the maximum Eigen value of the matrix) and *FV*^− 1^ is the next generator matrix. This leads to the following expression

$$ \begin{array}{l}{\mathrm{R}}_0 = \alpha \beta \Omega \left\{\frac{1}{\left(\upalpha +\upmu \right)\left({\uptheta}_{\mathrm{I}}+{\updelta}_{\mathrm{I}}+\upmu \right)+\uptau \left(\upalpha +\upmu \right)}+\frac{\uptau}{\left(\upalpha +\upmu \right)\left({\uptheta}_{\mathrm{I}}+{\updelta}_{\mathrm{I}}+\upmu \right)+\uptau \left(\upalpha +\upmu \right)}\frac{\upeta}{{\mathrm{K}}_3}\right\}\\ {}\kern2.5em ={R}_1+{R}_2.\end{array} $$

Here, *R*_*1*_ and *R*_*2*_ reflect the continuation of infectious individuals from the community and from hospitals respectively. The epidemiological significance of the *basic reproductive ratio* R_0_ - which represents the average number of new cases generated by a primary infectious individual in a population where some susceptible individuals are at high risk and some infected individuals go to hospital - is that the Ebola pandemic can be effectively controlled by reducing the number of high-risk individuals and by decreasing peoples’ contact with hospitalized individuals with other individuals, be they relatives, health-care workers, people involved in burial processes, etc. This can bring the threshold quantity (*R*_*0*_) to a value less than unity. Biologically, this implies that the Ebola pandemic can be eliminated from the population when *R*_*0*_ < 1.

### Data sources

The epidemic data used in this study wacollected by the WHO during the current outbreak; the data is available at http://apps.who.int/ebola/en/current-situation/ebola-situation-report. The CDC analyzed this data and proposed that there is under-reporting of the cases. They estimate that the true number of cases is 2.5 times more than the ones reported [19]. We use the raw or uncorrected data from the WHO, and then the corrected data from the CDC from May 1st, 2014 until October 1st, 2014 to estimate R_0_ for Liberia and Sierra Leone.

### Parameter values

Based on previous studies, the infection period for EVD is six days. The latent period of EVD is between two and seventeen days with a mean of ten days. The generation time of the disease is about 16.6 days. So, the values are 1/*θ*_*I*_ = 6 and 1/*α* = 10. The parameter *η* is the relative transmissibility of hospitalized individuals compared to infected individuals. The value of *η* < 1 would indicate that isolation in hospitals is effective. The value *η* > 1 would indicate ineffectiveness of transmissibility in hospitals. Such would be the situation in some developing countries. For West Africa, the value of *η* lies between 0.6 and 0.8. The parameter *ψ*_*H*_ accounts for infection that arises from the individuals involved in burial processes as well as health care workers. This indicates that high-risk individuals are more likely to get infected as compared to low-risk individuals. Its value lies between 1.5 and 2. The average time from hospitalization to recovery and from infection to recovery is five and 10 days respectively. Moreover only 45% of the infected and 50% of hospitalized individuals recover from the disease. The death rates of the infected and hospitalized individuals, *δ*_*I*_ and *δ*_*H*_, are 0.10 and 0.5, respectively. Of the infected individuals, 80% are hospitalized, and on average, hospitalization lasts four to five days after getting infected. These parameter values are taken from [11,12,20,21]. The assigned values are summarized in Table 1 below. All rates are defined per day except for μ.

### Estimation scheme

In order to calculate R_0_, we use parameter values for Model 1 as stated in Table 1 in the previous section. The estimates for several of the model parameters used in model (1) have been obtained from existing studies on EVD. The effective contact rate *β*, which is a measure of the rate at which contact between an infected and a susceptible individual occurs, and the probability that such contact will lead to an infection, is extremely difficult to determine directly. Therefore, we adopt an indirect approach, similar to previous studies such as [22] and [23], by first finding the value of parameter *β* for which Model 1 has the best agreement with the epidemic data, and then using the resultant parameter values to estimate R_0_.

Furthermore, we require knowledge of the initial conditions to be used for simulation of the trajectories of Model 1. It is possible to consider the initial conditions (*S*_*L*_*(0), S*_*H*_*(0), E(0), I(0) H(0),R(0)*) as model parameters, along with the effective contact rate and estimate values for all parameters. Such a technique, however, produces slightly unreliable results. This is explained by the fact that the available epidemic data is restricted to the reported cumulative case number, while the optimization scheme that we employ produces estimates for six variables.

There are thus too many degrees of freedom and the ‘best-fit’ may result in unrealistic estimates for the initial conditions used. We use the number of cases first recorded as the initial conditions and restrict ourselves to optimizing only the effective contact rate. The following initial conditions were used; *S*_*H*_(0) = 20000, *S*_*L*_(0) = one million, *E*(0) = 15, *I*(0) = 10, *H*(0) = 0, and *R*(0) = 0. This initial data is based on the fact that the total population in the region that was under threat of Ebola was one million. Among this population, 2% are at high-risk. Initially there were only a few exposed and infected individuals, and no hospitalized or recovered individuals.

In the following section we employ ordinary least squares (OLS) estimation to estimate the parameter *β* by minimizing the difference between predictions of Model 1 and the epidemic data. This is implemented by using the *fminsearch* function in the built-in optimization toolbox in MATLAB. The function *fminsearch* searches the minimum of a function of several variables. An initial guess of the variables is provided. Then, by using that guess, it searches for the values that are local minimizers of the function. This allows us to estimate the parameter *β* in order to calculate R_0_.

## Results

**Figure 2:**
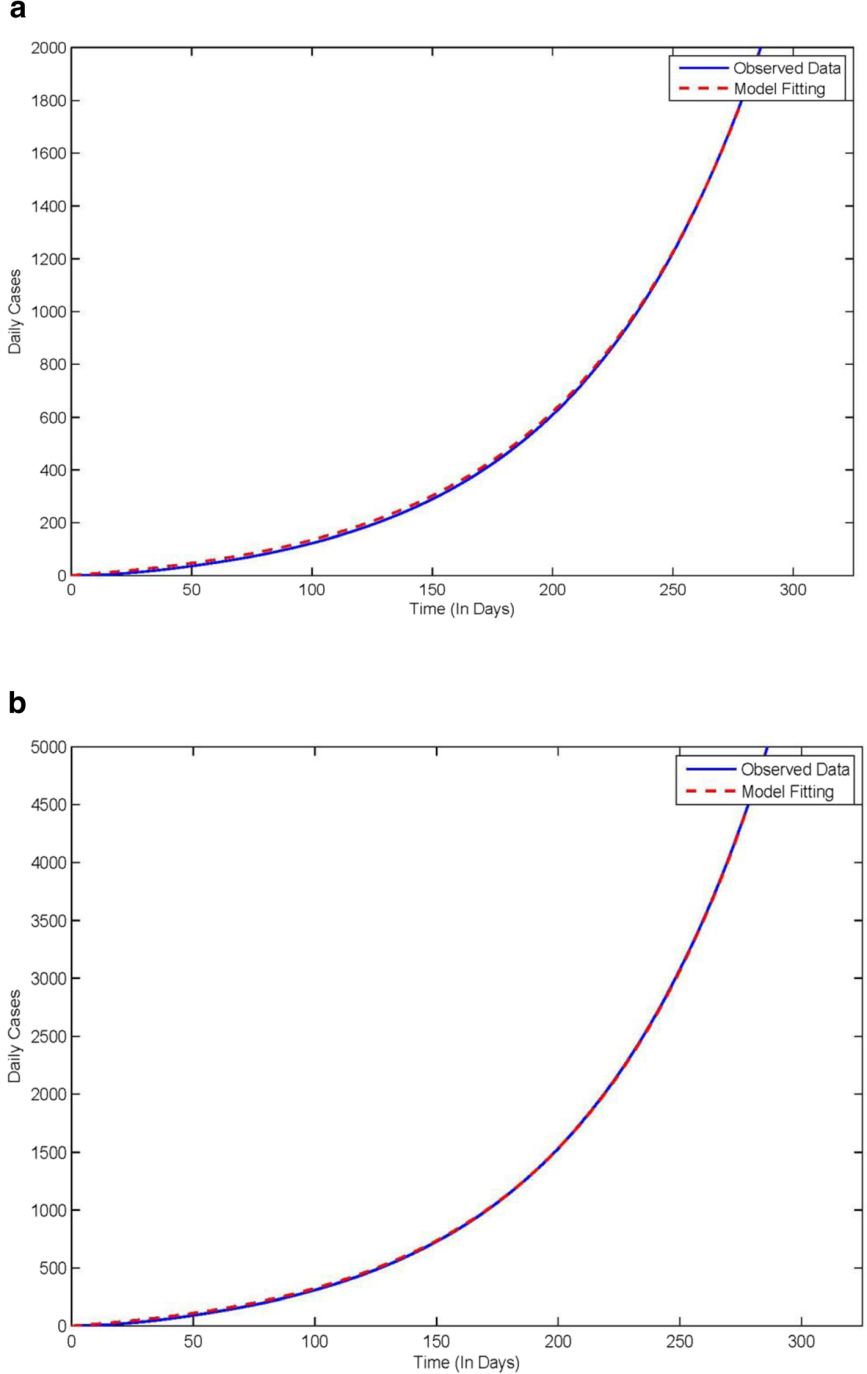
**Shows data fitting using model for Ebola cases in Liberia. a**: Uncorrected cases **b**: Corrected cases.

As described in previous section, we fitted the two different data sets, with and without correction for under reporting, for Liberia and Sierra Leone. Figure 2a shows the fit for the uncorrected Liberia data until October 1st, 2014 for which *β* is 0.371 and R_0_ is 1.757. Figure 2b shows the fit for the corrected Liberia data until October 1st, 2014 for which *β* is 0.3906 and R_0_ is 1.9.

**Figure 3:**
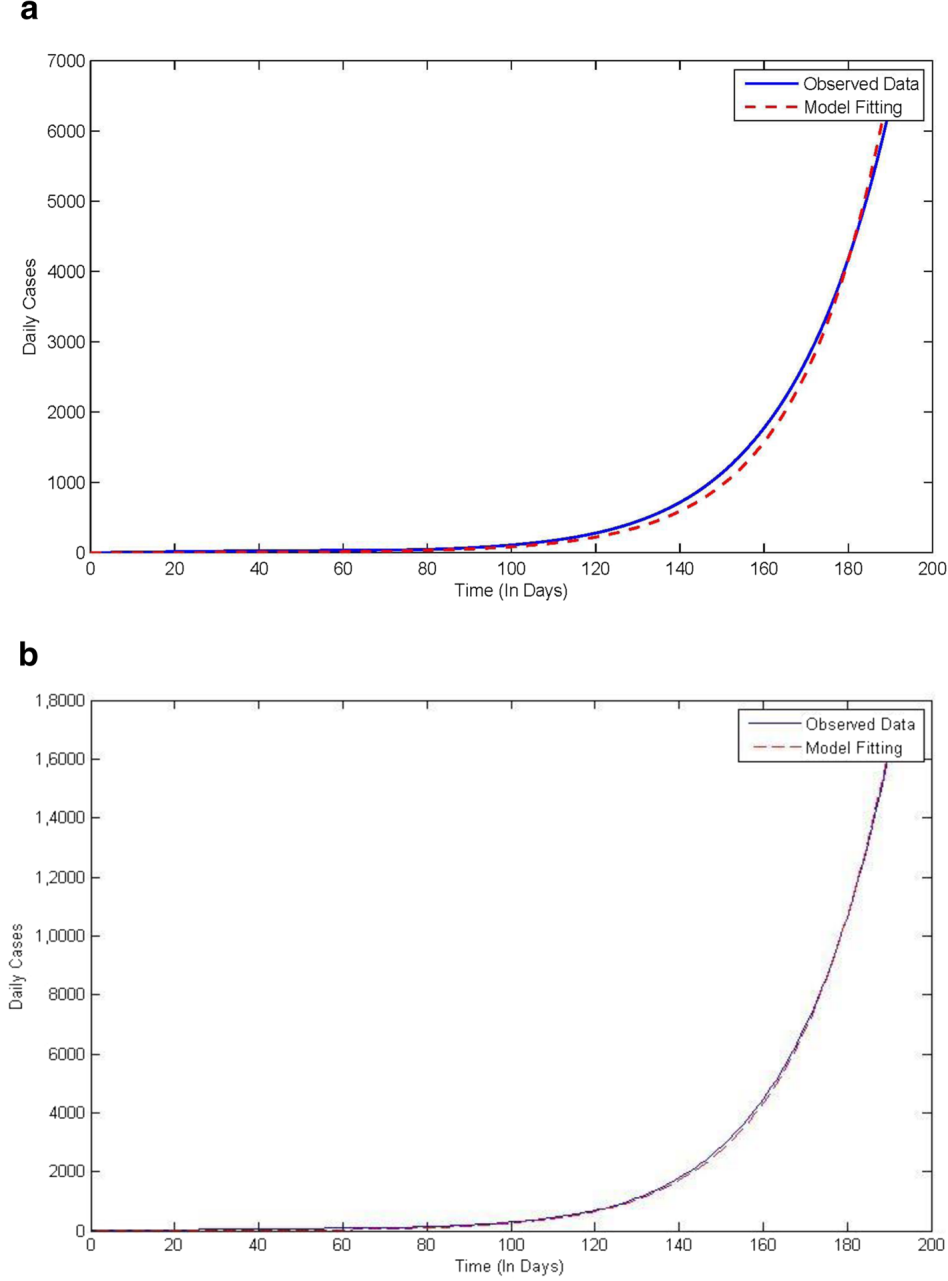
**Shows data fitting using mathematical model for Sierra Leone. a**: Uncorrected cases. **b**: Corrected cases.

For Sierra Leone, *β* is 0.361 and R_0_ is 1.492, as shown in Figure 3a for the uncorrected case. After correcting for under-reporting, *β* becomes 0.344 and R_0_ is 1.3682, as shown in Figure 3b.

**Figure 4:**
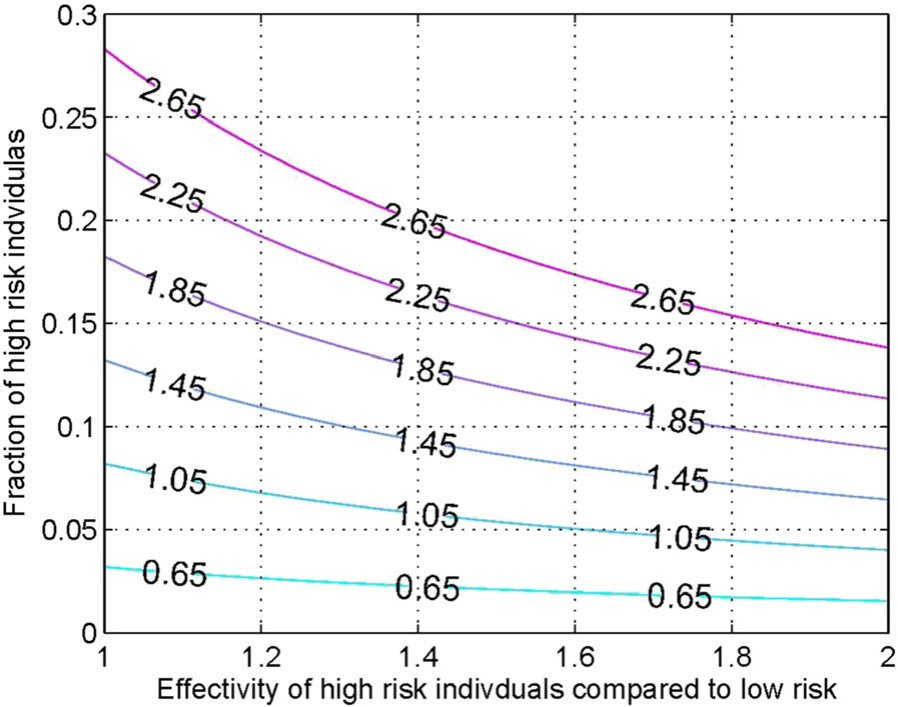
**Contour graph of the effectiveness of high-risk individuals versus a fraction of individuals at high risk.**

Finally, we study the variation in R_0_ with the relative risk of the highly susceptible population group and its percentage in the total susceptible population group. We note that less than 10% of the susceptible population should be in the high-risk group in order to bring R_0_ to less than 1. This is shown in Figure 4 below.

**Figure 5:**
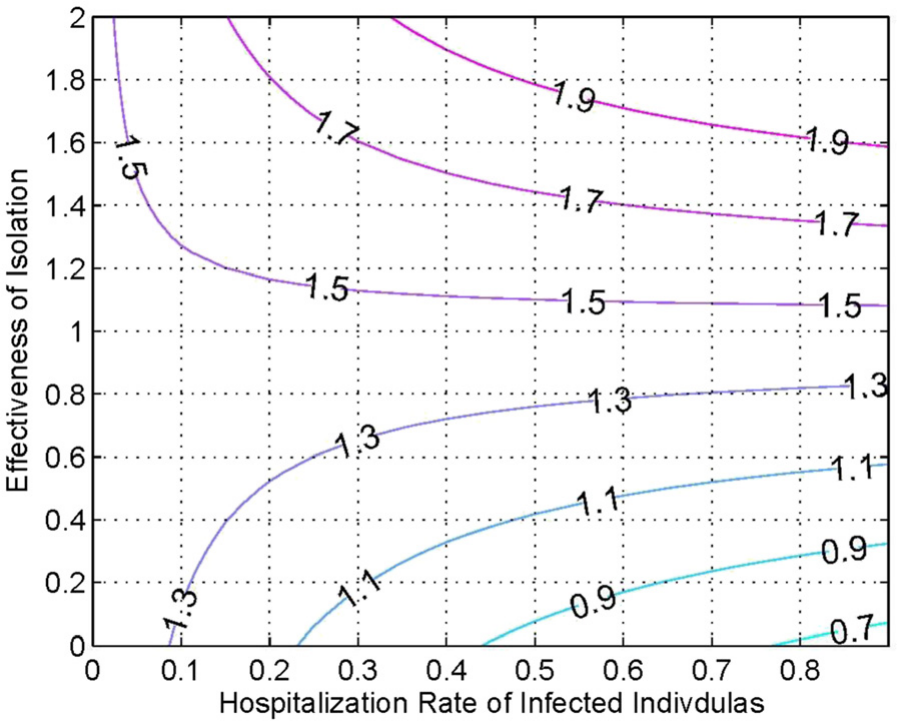
**Contour graph of the hospitalization rate versus the effectiveness of isolation.**

We also use our model to study the effects of isolation on R_0_. As shown in Figure 5 we note that not only does isolation have to be very effective, reducing the infectivity to less than 0.25, but at the same time around 45% or more of the population has to be isolated in order to bring R_0_ to a value less than 1.

## Discussion and conclusion

We have developed a model for the transmission dynamics of EVD, incorporating the important factor of the individuals who are at a much greater risk of contracting the disease than the general population in the affected areas. These include frontline health-care workers, family members of EVD patients, and individuals involved in the burial process of deceased EVD patients.

Using data from the WHO and CDC, we have calculated estimates for Liberia and Sierra Leone for the ongoing EVD outbreak. The values are significantly above 1, indicating the severity of the disease. The estimated values for Liberia are consistent with published estimates for the current outbreak [11,20,24], while those for Sierra Leone are consistent with Nishiura et al., however another recent study [8] estimated that R_0_ in this country is 2.53, which is significantly greater.

We have also looked at the effect of interventions to control the outbreak. In absence of any vaccine or medication for EVD, the only control measure available is isolation. Ideally during isolation strict control should be in place so that the isolated individuals do not transmit the disease. However, in practice, there is a non-zero risk of transmission from isolated individuals. Our analysis suggests that in order for R_0_ to reduce to less than 1, the transmission rate of the isolated individuals should be less than one quarter of that for the non-isolated. This means that strict protocols should be followed at treatment facilities. Further analysis of the model also leads to the conclusion that the fraction of high-risk individuals has to be controlled and must be brought to less than 10% of the overall susceptible population in order to bring R_0_ to less than 1 and hence control the outbreak.

Our model is an attempt to capture the most important features of the transmission dynamics of EVD. As an extension of this work, optimal, time-dependent strategies should be developed and advised to public health authorities in order to control the disease.

## Additional file

Additional file 1:
**Translation of the abstract into the six official working languages of the United Nations.**

## Abbreviations

EVD: Ebola virus disease
CDC: Centers for disease control and prevention
WHO: World Health Organization
SEIR: Susceptible-exposed-infected-recovered
ODE: Ordinary differential equation
OLS: Oordinary least squares
