# Supplementary material for: Estimating the basic reproductive ratio for the Ebola outbreak in Liberia and Sierra Leone
Source: Infect Dis Poverty. 2015 Feb 24;4:13. doi: 10.1186/s40249-015-0043-3 (PMC4347917; doi:10.1186/s40249-015-0043-3)
Supplement: Additional file 1: — Translation of the abstract into the six official working languages of the United Nations. [file 40249_2015_43_MOESM1_ESM.pdf]

Translation of the abstract into the six official working languages of the United Nations

تقدير النسبة التناسلية الأساسية لتفشي الإيبولا في ليبيريا وسيراليون

عدنان خان، ماهيم نافيد، محمد در-إي-أحمد، مودسّر عمران

نبذة

معلومات عامة: ظهر مرض فيروس الإيبولا من جديد كأزمة صحية عامة رئيسية في إفريقيا، مع حالات معزولة تمت ملاحظتها أيضا على الصعيد العالمي، خلال الانتشار الحالي. بهدف تقدير النسبة التناسلية الأساسية R_0، وهو مقياس لخطورة الانتشار.

الطرق: طورنا نموذج تأكيد النوع (قابل – معرّض – مصاب - متعاف)، واستخدمنا البيانات في مراكز السيطرة على الأمراض والوقاية من تفشي الإيبولا في ليبيريا وسيراليون. تتوفر مجموعتان من البيانات المختلفة: مجموعة بيانات أولية مبلّغ عنها ومجموعة بيانات مصحّحة (كبيانات مراكز السيطرة على المرض التي يُشتبه بأنه لا يتم الإعلان عنها بشكل كامل).

النتائج: باستخدام نموذج انتقال المعادلة التفاضلية المؤكدة العادية لوباء الإيبولا، جاءت نتيجة النسبة التناسلية R_0 لليبيريا على التوالي 1.757 و 1.9 لبيانات الحالة المصحّحة وغير المصحّحة. في سيراليون، جاءت نتيجة النسبة التناسلية R_0 على التوالي 1.492 و1.362 لبيانات الحالة المصحّحة وغير المصحّحة. في كل من الحالتين التي عالجناها، كان تقدير النسبة التناسلية الأساسية أكبر، في البداية، من الوحدة التي تؤدي إلى تفشي الوباء.

الخلاصة: حصلنا على تقديرات عالية لقيمة R_0 المرتبطة بإيبولا التي انتشرت في العام 2014، وأظهرت أن هناك توافقا وثيقا مع تقديراتنا للـR_0. كما أظهر تحليل نموذجنا أيضا أن هناك حاجة إلى العزلة الفعالة، بما أن معدل التواصل في العزلة هو أقل من ربع النسبة من السكان المصابين غير المعزولين، ويجب أن تنخفض نسبة الأفراد ذات المخاطر العالية إلى أقل من 10٪ من مجموع السكان المعرّضين، من أجل أن تصل قيمة R_0 إلى أقل من 1، وبالتالي السيطرة على انتشار المرض.

Translated from English version into Arabic by Sophiechammas, through

TRANSLATORS
WITHOUT BORDERS

估测利比里亚与塞拉利昂埃博拉疫情的基本复制率

Adnan Khan, Mahim Naveed, Muhammad Dur-e-Ahmad, Mudassar Imran

摘要

引言：当前的埃博拉疫情已再次成为非洲的重大公共卫生危机，且有分布于全球各地的个别病例。基本复制率（basic reproductive ratio，R_0）是衡量疫情严重程度的指标。

方法：本研究利用利比里亚和塞拉利昂疾控中心的埃博拉疫情数据，建立了 1 个“易感-暴露-感染-康复”（susceptible-exposed-infected-recovered，SEIR）决定模型。有两个不同的数据集，一份是原始报告数据，另一份是订正数据（疾控中心漏报疑似病例）。

结果：使用决定性常微分方程传播模型研究埃博拉疫情，得到的基本复制率是：利比里亚基于原始数据的是 1.757，基于订正数据的是 1.9；塞拉利昂分别是 1.492 和 1.362。在两个案例中，个案基本复制率大于整体的，从而引起疫情爆发。

结论：本研究获得的 R_0 值与 2014 年埃博拉疫情相关的有效 R_0 值一致。模型分析显示，有必要进行有效的隔离，当隔离的接触率低于感染且未隔离人群接触率的 1/4，并且高危个体的比例降到整个易感群体的 10% 以下，可使基本复制率小于 1，就能控制疫情。

Translated from English version into Chinese by Yin Jian-hai, through

CHINESE CENTER FOR DISEASE CONTROL AND PREVENTION
NATIONAL INSTITUTE OF PARASITIC DISEASES

Estimation du taux de reproduction de base pour l'épidémie de fièvre d'Ébola au Liberia et au Sierra Leone

Adnan Khan, Mahim Naveed, Muhammad Dur-e-Ahmad, Mudassar Imran

Résumé

Contexte: Le virus d'Ébola suscite actuellement une nouvelle crise majeure de santé publique en Afrique et des cas isolés sont identifiés partout dans le monde.

Méthodes: Afin d'estimer le taux de reproduction de base R_0 qui mesure la gravité de l'épidémie, nous avons mis au point un modèle déterministe de type SEIR (susceptible-exposé-infecté-remis) et utilisé les données des Centers for Disease Control and Prevention (CDC) pour l'épidémie de fièvre d'Ébola au Liberia et au Sierra Leone. Nous disposions de deux ensembles de données différents: des données brutes rapportées et des données corrigées (car les CDC suspectent qu'il pourrait y avoir une sous-déclaration).

Résultats: En utilisant comme modèle de transmission pour l'épidémie d'Ébola une équation différentielle ordinaire déterministe, nous avons trouvé un rapport de reproduction de base R_0 de 1,757 avec les données de cas corrigées et 1,9 avec les données non corrigées pour le Liberia. Pour le Sierra Leone, nous avons trouvé des valeurs de R_0 de 1,492 avec les données corrigées et 1,362 avec les données non corrigées. Dans les deux cas examinés, l'estimation du rapport de reproduction de base était initialement supérieure à 1, ce qui correspond à une propagation de l'épidémie.

Conclusion: Nous avons obtenu de bonnes estimations de la valeur de R_0 associée à l'épidémie de virus d'Ébola de 2014 et montré qu'il existait une concordance étroite entre nos estimations de R_0. L'analyse de notre modèle a également montré qu'un isolement efficace était nécessaire, avec un taux de contacts à l'isolement inférieur à un quart de celui de la population infectée non isolée, et que la fraction de sujets à haut risque devait être réduite à moins de 10 % de la population susceptible totale pour faire baisser R_0 en dessous de 1 et juguler ainsi l'épidémie.

Translated from English version into French by Suzanne Assenat, through

TRANSLATORS
WITHOUT BORDERS

Оценка основного репродуктивного коэффициента вспышки эпидемии лихорадки Эбола в Либерии и Сьерра-Леоне

Аднан Хан, Махим Навид, Мухаммад Дур-и-Ахмад, Мудассар Имран (Adnan Khan, Mahim Naveed, Muhammad Dur-e-Ahmad, Mudassar Imran)

Отрывок

История вопроса: Возрождение вирусного заболевания Эбола поразило Африку как глобальный кризис здоровья населения, единичные случаи которого, в ходе текущей вспышки, фиксируются по всему миру. Для оценки основного репродуктивного коэффициента R_0, который является критерием остроты вспышки.
Методы: Текущей эпидемии в Либерии и Сьерра-Леоне, мы разработали детерминистическую модель SEIR (восприимчивый-в инкубационном периоде-инфицированный-выздоровевший) и использовали данные из Центров по профилактике и контролю заболеваемости. В распоряжении имеются два различных набора данных: один - с первичными зарегистрированными данными, а другой - с откорректированными данными (поскольку Центры по профилактике и контролю заболеваемости допускают вероятность занижения отчетных показателей).
Результаты: Используя детерминистическую модель передачи с обыкновенным дифференциальным уравнением, основной репродуктивный коэффициент R_0 для Либерии составил 1,757 и 1,9 для откорректированных и неоткорректированных данных по случаям соответственно. По Сьерра-Леоне R_0 составил 1,492 и 1,362 для откорректированных и неоткорректированных данных по случаям соответственно. В каждом из двух рассмотренных случаев предполагаемая величина основного репродуктивного коэффициента изначально была выше, чем единица, ведущая к вспышке эпидемии.
Заключение: Мы получили обоснованные расчеты значения R_0, связанного со вспышкой эпидемии лихорадки Эбола в 2014 году, и продемонстрировали близкое совпадение результатов наших оценок коэффициента R_0. Анализ нашей модели также показал необходимость проведения эффективной изоляции. Причем коэффициент контактов при изоляции должен составлять менее одной четверти для инфицированного неизолированного населения, а доля индивидов с высоким уровнем риска должна быть сведена к менее чем 10% от общего количества уязвимого населения, чтобы значение R_0 составило менее 1, и следовательно, появилась возможность взять под контроль вспышку эпидемии.

Translated from English version into Russian by tatiana_com, through

TRANSLATORS
WITHOUT BORDERS

Cálculo de la tasa básica de reproducción para el brote de Ebola en Liberia y Sierra Leona

Adnan Khan, Mahim Naveed, Muhammad Dur-e-Ahmad, Mudassar Imran

Sumario
Antecedentes: La enfermedad por el virus del Ebola ha resurgido como una gran crisis de salud pública en África y, durante el brote actual, también se observan casos aislados nivel mundial. Para estimar la tasa básica de reproducción R_0, que es una medida de la gravedad del brote.
Métodos: Hemos desarrollado un modelo determinista tipo SEIR (susceptible-expuesto-infectado-recuperado) y hemos utilizado datos de los Centros para el Control y la Prevención de Enfermedades (CDC), para el brote de Ebola en Liberia y Sierra Leona. Hay disponibles dos conjuntos diferentes de datos: uno con los datos brutos reportados y otro con los datos corregidos (dado que el CDC sospecha que muchos casos no se reportan).
Resultados: Al emplear un modelo determinista de transmisión basado en la ecuación diferencial ordinaria para la epidemia de Ebola, la relación de reproducción básica R_0 para Liberia resultó ser de 1,757 y de 1,9 para los datos

de casos corregidos y sin corregir, respectivamente. Para Sierra Leona, R_0 resultó ser de 1,492 y de 1,362 para los datos de casos corregidos y sin corregir, respectivamente. En cada uno de los dos casos que consideramos, el cálculo aproximado de la tasa básica de reproducción fue inicialmente superior a la unidad, lo que conduce a un brote epidémico.

Conclusión: Obtuvimos sólidos cálculos aproximados para el valor de R_0 asociado con el brote de Ebola de 2014, y mostramos que existe un estrecho acuerdo entre nuestros cálculos aproximados de R_0. El análisis de nuestro modelo también mostró que es necesario un aislamiento eficaz, con la tasa de contacto en el aislamiento de menos de una cuarta parte de la tasa de las personas infectadas sin aislamiento, y que la fracción de individuos de alto riesgo debe reducirse a menos del 10% de la población susceptible general, con el fin de reducir a menos de 1 el valor de R_0 y, en consecuencia, controlar el brote.

Translated from English version into Spanish by María Diehn, through

TRANSLATORS
WITHOUT BORDERS
